# Association between hypertension, obesity and dietary intake in post-menopausal women from rural Zambian communities

**DOI:** 10.4102/hsag.v26i0.1496

**Published:** 2021-08-13

**Authors:** Joseph M. Chalwe, Upasana Mukherjee, Christa Grobler, Saidon H. Mbambara, Wilna Oldewage-Theron

**Affiliations:** 1 Department of Health Sciences, Faculty of Applied and Computer Sciences, Vaal University of Technology, Vanderbijlpark, South Africa; 2 Department of Nutritional Sciences, Faculty of Human Sciences, Texas Tech University, Lubbock, Texas, United States of America; 3 Department of Biomedical Sciences, Tropical Diseases Research Centre, Ndola, Zambia; 4 Department of Nutritional Sciences, Faculty of Health Sciences, Texas Tech University, Lubbock, Texas, United States of America; 5 Department of Nutrition and Dietetics, Faculty of Health Sciences, University of the Free State, Bloemfontein, South Africa

**Keywords:** hypertension, obesity, diet, post-menopausal, rural, Zambia

## Abstract

**Background:**

Amongst the cardiovascular risk (CVR) factors, hypertension (HT) and obesity appear to be prominent in post-menopausal women. The underlying mechanisms of HT development in menopause are not fully understood.

**Aim:**

To determine the association between HT, obesity and dietary intakes in post-menopausal women from rural Zambia.

**Setting:**

This study was conducted in Twatasha Compound of Kitwe and Ndeke Community of Ndola.

**Methods:**

Blood pressure (BP), weight, height and dietary intakes (24-h recall) were measured in 153 women (> 50 years) from households. The South African Hypertension Society (SAHS), the World Health Organization (WHO) and estimated average requirements (EARs) guidelines were followed for HT, obesity and dietary intake definitions, respectively. Statistical Package for the Social Sciences (SPSS) version 26 was used for descriptive and inferential statistical analyses.

**Results:**

Prevalence of HT was 70%, whilst 37.25% and 28.10% of the participants were overweight and obese, respectively. The median interquartile range (IQR) dietary intakes showed inadequate intakes for most nutrients, except for carbohydrates (170 g [133; 225]). The total fat intake represented 14% of total energy intake. All median fatty acid intakes and sodium intakes (409 mg [169; 662]) were below the recommended levels. Only body mass index (BMI) correlated with HT (*r* = 0.268; *p* = 0.001).

**Conclusions:**

Despite low dietary intakes, an alarming prevalence of HT and obesity was found in our population. Hormonal changes, a high energy-dense diet and poor treatment adherence, may be possible underlying factors. We recommend measures to better manage HT as a CVR factor.

**Contribution:**

This article supplements evidence on the prevalence of obesity-related hypertension in post-menopausal women and the link to dietary intake.

## Introduction

Hypertension (HT), also known as high blood pressure (BP), is a critical medical condition and a major modifiable risk factor for cardiovascular disease (CVD). It affects over 1.1 billion people globally and is a significant cause of premature deaths (World Health Organization [WHO] 2019). The burden of HT is growing in sub-Saharan Africa (SSA), especially in the elderly population (65 years and older). The prevalence of HT and its complications increase with age and have been reported to be higher in women than men (Ahmad & Oparil 2017; Guwatudde et al. 2019; National Department of Health (NDOH) 2019; WHO 2019). Although the mechanisms leading to its development are not fully understood, several factors have been reported. These include leptin, the sympathetic nervous system (SNS), the renin–angiotensin–aldosterone system (RAAS), the natriuretic peptide (NP) system and compression of the renal system from the excess accumulation of fat to mention a few (Chrysant 2019). Obesity is also a risk factor for CVD, type 2 diabetes mellitus (T2DM) and HT. The prevalence of overweight and obesity is increasing around the world (over 1 billion) and projected to become a significant epidemic with serious economic, social and health challenges in various population groups (Chrysant 2019; WHO 2020). A number of causal factors for the development of obesity in women especially have been described including menopause (ageing), adoption of a less active lifestyle and an increased energy dense diet amongst others. Not only has diet been associated with obesity, but evidence also exists for the important role that dietary factors play in both primary and secondary prevention of HT (Schwingshaki et al. 2017). Excess weight, lack of physical activity and high sodium intakes have been established as the main dietary and lifestyle factors associated with HT (Poggiogalle et al. 2019). Numerous research studies have investigated the effect of various food groups, such as whole grains, nuts, pulses, fruits, dairy and eggs, as well as flesh foods (Ndanuko et al. 2016; Schwingshacki et al. 2017) and nutrients, for example, sodium, potassium (Ndanuko et al. 2016), calcium, magnesium (Villa-Etchegoyen et al. 2019) and various amino acids (Poggiogalle et al. 2019) on BP. Recently, there has been a growing interest to investigate the potential effect of specific nutrients on BP (Poggiogalle et al. 2019).

Menopause is a natural phase in a woman’s life caused by a decrease or cessation in the production of oestrogen that presents several physiologic, metabolic and mental well-being challenges as well as most importantly an increased risk for developing CVD (Carmel 2020; Gonçalves et al. 2016). A well-established association between obesity and HT has been reported in various population groups, especially in developed countries (Hossain et al. 2019; Manzoor & Zaib 2019; Shuger et al. 2008). Assessing the correlation between HT and obesity has significant implications on the health sector of developing countries, where the burden of HT and obesity is growing rapidly (Hossain et al. 2019; Misra, Jayawardena & Anoop 2019; Neupane et al. 2014). In addition, exploring these associations distinguished by gender, age and socioeconomic status is essential to understand the relationships across different settings to determine whether they are possibly biological or environmental (Hossain et al. 2019). Gaining insight into the association between HT and obesity as well as the underlying mechanisms in the pathogenesis of obesity-related HT is crucial for the effective treatment of HT of obese individuals (Chrysant 2019). There is inadequate information on the cardiovascular health of the elderly in African populations, which partially contributes to the minimal attention invested in this group (Aboderin 2010; Audain et al. 2017; Bosu et al. 2019; Chirwa & Kalinda 2016). Hypertension has been reported in rural Zambians, double that of the urban population (Rush et al. 2018). Moreover, Chirwa and Kalinda (2016) report that there is sparse information or statistics on the elderly population in Zambia and their health status despite being amongst the poorest households in the country. For this reason, this study was designed and conducted in post-menopausal women from two rural Zambian communities to assess the association between HT, obesity and their dietary intakes. To the best of our knowledge, there is no study or report in these two communities that investigated the link between these parameters.

## Research methods and design

### Study design, setting and population

A cross-sectional survey design was used. A power calculation based on 95% confidence level (Creative-Research-Systems 2020) was used to determine a sample size of 372 women needed for this study. However, a convenience sampling method was used and only 153 participants from household settings were recruited because of a cholera outbreak in the country and a subsequent temporary ban on public gatherings. The study involved voluntary participants who met all the inclusion criteria, namely aged > 50 years, post-menopausal, black (race), gave informed consent to participate and were residents of two rural communities: Twatasha Compound in Kitwe and Ndeke Community in Ndola of Zambia. There were no exclusion criteria. The data were collected between April 2018 and December 2018.

### Data collection

#### Blood pressure measurements

Socio-demographic and medical history data were collected but not included in this report. The subjects were requested to sit quietly for at least 5 min in a chair with a back support, feet on the floor and arm supported at heart level before the measurements. The Tensoval® duo control monitor was wrapped firmly around the right arm wrist consistently to measure systolic and diastolic BP readings in duplicate on two different days by a Health Professions Council of Zambia (HPCZ)-registered nurse. For accuracy, the mean systolic and mean diastolic readings were then determined and recorded. Hypertension was defined according to the South African Hypertension Society (SAHS) guidelines at ≥ 140/90 mmHg. These guidelines were used to further divide the elderly population into the categories: normal, optimal, high normal and HT grade (1–3) (Table 1).

**TABLE 1:** Overview of the respondent’s hypertension categories according to the South African Hypertension Society 2019 guidelines.

Blood pressure	Systolic (mmHg)	Diastolic (mmHg)	*N* = 153	Percentage (%)
Normal	< 120	< 80	14	9.15
Optimal	120–129	< 80	13	8.50
High normal	130–139	80–89	15	9.80
Hypertension				
Grade 1	140–159	90–99	40	26.14
Grade 2	160–179	100–109	28	18.30
Grade 3	> 180	> 110	41	26.80

#### Anthropometric measurements

The anthropometric measurements including weight and height were measured by an HPCZ-registered nurse and trained fieldworker using a calibrated Philips electronic scale (Amsterdam, The Netherlands), model HF350 (135 kg/100 g) and a Scales 2000 (Durban, South Africa) stadiometer. Stature was recorded as the height from the floor to the top of the head. It was ensured that the floor surface was even and firm. The participants were measured barefoot. The participant stood straight up, heels together, arms hanging down sides (palms facing thighs). The platform of the stadiometer was dropped until it made contact with the top of the head. The results were recorded to the nearest 0.1 cm. The participants were weighed with as little clothes as possible and without shoes. It was ensured that the scale measured zero before the participant stepped on. The results were recorded to the nearest 0.1 kg (Gibson 2005) (Beurer, Germany). The body mass index (BMI) was then calculated as weight divided by height squared (kg/m^2^) (Nuttall 2015; WHO 2020). World Health Organization guidelines were also used to distribute our population into the underweight, normal weight, overweight and obesity classifications (WHO 2020).

#### Dietary intake measurements

The 24-h dietary recall method was used for dietary intake assessments as it is an easy, quick and inexpensive method by a nutritionist in a one-on-one interview with respondents. The 24-h recall was collected on a week day before the survey date. The validated five-step multiple pass method was used (Gibson et al. 2015) in a one-on-one interview with the participants, assisted by a trained fieldworker. Food models were used to estimate portion sizes. Dietary intake data were analysed by a nutritionist using the software Food Finder, version 3, developed by the Medical Research Council of South Africa (MRC).

### Data analysis

The raw data of the BP readings, BMI, dietary intakes and age were all captured on a Microsoft Excel spreadsheet, and then the means ± standard deviations (SDs) were calculated. Raw data were cross-checked with captured data to ensure reliability. The IBM SPSS Statistics ® version 26 was used for descriptive and inferential statistical analyses. Means and SDs were used for normally distributed data and medians (interquartile range [IQR]) for data that were not normally distributed. Interquartile ranges were used to measure the dispersion of the data. Dietary data intake was not normally distributed; therefore, median and IQR were calculated to describe dietary intake. Estimated energy requirements (EERs) were calculated for each participant. The physical activity level (PAL) was considered to be moderate as the women worked in small farms during the day. The formula used for calculating the EER was:

EER = (354–[6.91 × age] + PAL × [9.36 × weight + {kgs}] + [726 × height {m}]).

The formula used for calculating required protein intake was – 0.8* body weight in kilograms. The medians of the nutrient intakes were then compared to the EARs for elderly females (51–70 years and > 70 years). The calculated EER and required protein were used to estimate energy and protein intake for each individual. Frequencies were used to determine the percentage of the sample with inadequate (< 100% EAR/EER/FAO & WHO guidelines) or too high (FAO & WHO) intakes. A Spearman’s correlation analysis was performed between the participants’ HT, BMI and dietary intake because it is more appropriate for ordinal data.

### Ethical considerations

The Tropical Diseases Research Centre (TDRC) Ethics Review Committee (STC/2017/19) and the National Health Research Ethics Board (NHREB), University Teaching Hospital, Zambia ethically approved this study. Permission to undertake the study was obtained from the community leaders of the two communities after explaining the purpose, objectives and methods of the study. Anonymity and confidentiality were ensured during the collection of the anthropometric data. Weighing was performed in a private room where only the participant and the fieldworker were present. Participants with alarming BP readings were referred to the clinic.

### Validity and reliability

An HPCZ-registered nurse and trained fieldworker recorded all anthropometric measurements according to standardised procedures (Gibson 2005) using a calibrated Philips electronic scale (Amsterdam, The Netherlands), model HF350 (135 kg/100 g) and a Scales 2000 (Durban, South Africa) stadiometer. Both weight and height measurements were taken twice to ensure that the recorded measurement was correct. A validated procedure was used for the 24-h recall questionnaire (Gibson 2005). Although a single 24 h dietary recall may not represent the dietary habits entirely and at least two interviews spaced over different days may represent a better picture (FAO 2010, 2018), only one 24-h recall was possible during the baseline survey because of a cholera outbreak. This limitation was partly overcome by the fact that a recent national study has shown that the traditional diets in Zambia are largely vegetarian and consist mainly of maize meal porridge and vegetarian side dishes that change throughout the year. This study has also found that the monotonous traditional diet has been largely unchanged over the past century (Harris et al. 2019). The 24-h recall questionnaire was prepared by following the guidelines in ‘Dietary Assessment Methodology’ (Thompson & Subar 2001). However, the validated five-step multiple pass method was used (Gibson et al. 2015). The pre- and post-measurements of BP were taken on different days to account for the BP fluctuations.

## Results

The mean ± SD age of the women was 58 ± 10 years. Overall, our study population was hypertensive with a prevalence of 70%. An interesting observation from Table 1 is that out of the 70%, 26.80% of the population fell into the HT grade 3 category.

A total of 37.25% of the population was overweight and 28.10% could be classified into the obesity grade 1 category. Although the median (IQR) BMI was 28.35 indicating overweight, the anthropometric results showed that 21% of the respondents were of normal weight. However, 37.25% and 39.86% of the respondents were overweight and obese, respectively (Table 2).

**TABLE 2:** Body weight status of the participants.

BMI	*N* = 153	Percentage (%)
Underweight (< 18.5)	3	1.96
Normal weight (18.5–24.9)	32	20.92
Overweight (25–29.9)	57	37.25
Obesity grade 1 (30–34.9)	43	28.10
Obesity grade 2 (35–39.9)	12	7.84
Obesity grade 3 (> 40)	6	3.92

BMI, body mass index.

**TABLE 3:** Nutrient intakes of the participants measured by once off 24-h recall.

Nutrients	EAR/EER†/AI‡/FAO guideline§	Median	IQR	Inadequate or too high¶ intakes (%)
**Energy (Kcal)**	2511 (2282; 2665)†	962.00	689; 1295	100.00
**Energy (KJ)**	10 498 (9539; 11 140)	4024.00	2888; 5409	
TE from carbs (%)	45–65	73.00	66; 83	
TE protein (%)	10–35	12.00	11; 16	
TE from fats (%)	25–35	14.00	11; 19	
**Carbohydrate (g)**	100	170.00	133; 225	13.7
**Dietary fibre (g)**	21‡	22.00	15; 29	49.0
**Protein (g)**	47 (0.66 g/kg body weight/day)	31.30	19; 45	88.2
Plant protein (g)		16.60	11; 22	
Animal protein (g)		0.00	0; 0	
**Total fat**	20–35§	14.00	9; 24	76.5
SFA (%TE)	< 10§	3.00	2; 26	5.9¶
TFA (%TE)	< 1§	0.01	0.00; 0.02	2.6¶
MUFA (%TE)	Balance (Total fat – SFA + TFA + PUFA)§	4.10	3; 8	60.8
PUFA (%TE)	6–11§	5.70	4; 9	64.7
Cholesterol (mg)	< 300§	12.00	0; 64	0.0¶
Sodium (mg)	≤ 2000	409.00	169; 662	9.1¶

EAR, estimated average requirement for women 51–70 years old; EER†, estimated energy requirement for women; AI‡, adequate intake; TE, total energy; FAO§, The Food and Agriculture Organization; IQR, interquartile range; MUFA¶, monounsaturated fatty acids; PUFA, polyunsaturated fatty acids; SFA, saturated fatty acids; TFA, trans fatty acids.

Total energy (TE) and macronutrients showed low intake when compared to EER and EAR, respectively. The acceptable macronutrient distribution ranges (AMDRs) for carbohydrate are 45% – 65%, 10% – 35% for proteins and 25% – 35% for fats. The participants consumed a high-carbohydrate and low-fat diet with a mean energy intake of 73%, and 12% for proteins and 14% from fats. Although carbohydrates showed a median intake much higher than EAR and dietary fibre showed adequate median intake, 14% of the participants did not have adequate carbohydrate intake, whilst almost half of the participants (49%) did not consume adequate fibre. Median intake of proteins with the majority of the participants (88%) did not meet required intake. The protein source was entirely from plant sources and none of the participants reported to consume any animal proteins. All the fatty acids, as well as sodium, showed intakes much lower than the recommended cut-off points.

### Correlation between the hypertension, body mass index and nutrient intakes (24-h recall)

The only significant positive relationship observed was between HT and BMI (*r* = 0.268; *p =* 0.001) (2-tailed) (Table 4).

**TABLE 4:** Spearman’s correlation between the different parameters and hypertension (*r*/*p*).

Hypertension	*r*	*p*
Body mass index	0.268	0.001*
Carbohydrates	0.085	0.981
Fibre	−0.015	0.111
Fat intake	0.124	0.474
Saturated fatty acids	0.141	0.548
Trans fatty acids	0.206	0.631
Monounsaturated fatty acids	0.117	0.569
Polyunsaturated fatty acids	0.059	0.910
Sodium	0.158	0.487

* , Correlation is significant at the 0.01 level (2-tailed).

## Discussion

In this study, the prevalence of HT of the women residing in the two rural Zambian communities was found to be alarmingly high. This is similar to the findings of Benjamin et al. (2017), who reported a high prevalence of HT in women over the age of 60 years in the United States of America (USA) (Ahmad & Oparil 2017; Benjamin et al. 2017). Gaziano et al. (2017) also reported similar results (58.4% in mean age 61.7 + 13.06 years) with statistically significant increases with age in 6281 elderlies residing in the rural Agincourt sub-district of Mpumalanga province, Republic of South Africa (RSA) (Gaziano et al. 2017). An alarming high prevalence of HT and pre-HT was also reported by Tripathy et al. (2017) in a 5127 population from Punjab, North India (Tripathy et al. 2017). Alcoholism, now called alcohol use disorder (AUD), diabetes and obesity have been reported as factors for HT in urban areas, whilst excessive salt intake (≥ 5 g/day) has been associated with HT in rural areas (Tripathy et al. 2017). Salt intake has also been linked to HT in previous studies and identified as a significant causal agent in its pathogenesis (Bhansali et al. 2014; Tripathy et al. 2017). Hormonal changes during menopause can lead to weight gain and make the BP more sensitive to salt in dietary intake which can sequentially lead to HT (Genazzani et al. 2018). However, in this study, a high prevalence of HT was observed despite low sodium intake. A recent review paper (Grillo et al. 2019) has found that an increased risk of HT is not only associated with high sodium intake, but also when there is significantly low sodium intake. Effects of a very low sodium intake ‘may be mediated by elevated renin-aldosterone activity and sympathetic neural activation’ (Grillo et al. 2019). A study amongst elderly women in South Africa also found a high prevalence of obesity and HT despite low sodium intake (Oldewage-Theron & Egal 2013). In addition, no association could be established between sodium intake and HT in our study population, but a positive (weak) significant correlation was found between BMI and HT, a result that is consistent to what was reported from Langa, the urban Prospective Urban Rural Epidemiology (PURE) study site in the Western Cape province of South Africa (Solomons, Kruger & Puoane 2018). Similar findings were reported from Bangladesh, India and Nepal (Hossain et al. 2019) as well as from Faisalabad District of Punjab province in Pakistan (Manzoor & Zaib 2019). This is not surprising because of the significant role played by overweight and obesity in the development of HT (Landi et al. 2018; Roka, Michimi & Macy 2015). These may perhaps be some of the reasons for the high prevalence of HT in our study population. Hypertension is the most prominent risk factor for CVD and the primary cause of death in women globally. Although the fundamental mechanisms and causes of HT are still not fully understood, a gender-related heterogeneity has been reported in the history of HT. Accumulating evidence also suggests that oestrogen protects women of childbearing age from developing HT. For this reason, as women age, the risk for developing HT and the related CVD outcomes increases because of the declining levels of oestrogen. The types of HT in women are also unique because of pregnancy, menopause and the use of contraceptives (Ahmad & Oparil 2017; Newson 2018).

Most of our population was overweight and a large percentage could be classified into the obesity grade 1 category. Our obesity findings are consistent with other studies in South Africa and the region (Gaziano et al. 2017). These findings are also consistent with a study that was carried out amongst 510 Rajbanshi women residing in the district of Darjeeling, West Bengal (Sinha, Mondal & Sen 2018). Similar findings were also reported in menopausal women from Montes Claros, Minas Gerais, Brazil (Gonçalves et al. 2016) as well as in post-menopausal women from Buffalo, New York, USA (Banack et al. 2019). Dietary habits, a sedentary lifestyle, changes in hormones, age, parity, education, age at menarche, tobacco use, occupation, monthly income, age at first and last pregnancy and a high energy dense diet have been reported to influence the prevalence of overweight and obesity in post-menopausal women (Carmel 2020; Gonçalves et al. 2016; Sinha et al. 2018). Maize or corn is the major staple food in Zambia. It is the main source of carbohydrates that is processed into a fine white powder called ‘mealie meal’ and is highly consumed. Mealie meal is cooked in a variety of ways and is a key component of a Zambian diet (Harris et al. 2019). However, no association was found with dietary intake. Hypertension, diabetes and obesity are more prevalent in women than men. The TE intakes were low when compared to EER, but the median carbohydrate intakes were very high, pointing to a carbohydrate-based diet that is consistent with maize being the staple food in Zambia. Total protein and fat intakes were also low. No proteins from meat, fish or poultry were consumed. These findings were consistent with a study undertaken amongst elderly women in South Africa (Oldewage-Theron & Egal 2013). A recent national study found that the Zambian population consumed a monotonous diet that were largely vegetarian and carbohydrate-based. The proportion of total dietary energy derived from cereals (carbohydrates) was more than 50% and mainly from maize. In addition, foods from animal origin and fats contribute less than 10% and 8% of dietary calories (energy), respectively (Harris et al. 2019). These findings that were consistent with a recent national study found that the Zambian population consumed a monotonous diet that was largely vegetarian and carbohydrate-based. The proportion of total dietary energy derived from cereals (carbohydrates) was more than 50% and mainly from maize. In addition, foods from animal origin and fats contribute less than 10% and 8% of dietary calories (energy), respectively (Harris et al. 2019), and Romieu et al. (2017) indicated that energy-dense foods and big portion sizes may have an obesogenic effect and studies have shown that women consuming a carbohydrate-based diet and refined foods were more at risk of obesity (Romieu et al. 2017). In many low- and medium-income countries, like South Africa, obesity coexists with undernutrition (Romieu et al. 2017). In addition, food insecurity is also associated with obesity, generally known as the food insecurity-obesity paradox (Dhurandhar 2016). The food insecurity prevalence in our community was 93% (Mukhurjee 2020). Food insecurity is often associated with inadequate dietary intakes as a result of consumption of more affordable and energy-dense foods instead of nutrient-dense foods (Walsh & Rooyen 2015).

### Strengths and limitations

A strength of our study is that, to our knowledge, this is the first report on the association between HT, BMI and dietary intake in these rural Zambian communities. In addition, this study contributes to current evidence on the risk factors of obesity-related HT. However, our study has some limitations. Firstly, only one 24-h recall assessment was conducted for each respondent which is not representative of usual dietary intakes. In addition, under-reporting of dietary intakes was found in a large percentage of the respondents. However, our results were consistent with a recent national study that found that per capita total dietary intake per day has been consistently lower than the daily recommended 8400 kJ since the 1990s (Harris et al. 2019). Blood pressure or Hypertension measurements were measured over a period of 6 months instead of ongoing measurements. Fluctuations in BP are a general occurrence as a result of many factors that we did not measure. Additionally, our findings should not be generalised as the sample is not statistically representative. Lastly, no HT mechanism parameters were measured.

### Recommendations

We recommend further studies with statistically significant numbers of diverse populations from both urban and rural settings as well as determining their RAAS parameter levels: angiotensin-converting enzyme (ACE), Angiotensin I (Ang I) (plasma renin activity [PRA]) and Angiotensin II (Ang II). Understanding the role of the pathophysiologic factors, leptin, the SNS, the RAAS and the NP in the pathogenesis of obesity-related HT, is key for successful treatment option development for HT in obese individuals as evidenced by other studies (Chrysant 2019; Landi et al. 2018; Roka et al. 2015), and future studies should examine these factors in this community. Conducting such studies will further the knowledge of BMI, the nutritional status and their role in the development of obesity-related HT which is vital in designing treatment options. It is recommended that CVD risk factors should be continually monitored and results used to develop and implement strategies for prevention, early detection through screening and treatment. Chrysant et al. (2019) reported that the most important treatment option for HT is a change in lifestyle, diet and exercise, and it is recommended that a tailored multi-strategy nutrition education programme be implemented in this community to raise awareness and empower the elderly women with nutrition knowledge and food preparation skills to make healthy food choices in future. Moreover, our study emphasises the urgent need for amended health efforts to increase the knowledge awareness of HT in rural communities and to alleviate poverty as a significant barrier to accessing quality healthcare (Jongen et al. 2019).

## Conclusion

The majority of our study population is hypertensive and overweight which puts them at risk for CVD. In addition, a mainly carbohydrate-based diet with poor nutrient intakes that further exacerbate the health status of these elderly women was observed. There is a paucity of data available on the health status of elderly women in Zambia and, like in other African countries, the elderly has been neglected with regard to public health interventions. However, the findings of this study point to a possible public health problem amongst elderly women in Zambia that is important information for policymakers.
